# Role-Driven Clustering of Stakeholders: A Study of IoT Security Improvement

**DOI:** 10.3390/s23125578

**Published:** 2023-06-14

**Authors:** Latifah Almalki, Amany Alnahdi, Tahani Albalawi

**Affiliations:** 1 Department of Computer Science, King Abdulaziz University, Jeddah 21589, Saudi Arabia; akalnahdi@kau.edu.sa; 2 Department of Computer Science, Imam Mohammad Ibn Saud Islamic University, Riyadh 11432, Saudi Arabia; tfalbalawi@imamu.edu.sa

**Keywords:** IoT security, clustering, stakeholders, role, decision making

## Abstract

This study aims to address the challenges of managing the vast amount of data generated by Internet of Things (IoT) devices by categorizing stakeholders based on their roles in IoT security. As the number of connected devices increases, so do the associated security risks, highlighting the need for skilled stakeholders to mitigate these risks and prevent potential attacks. The study proposes a two-part approach, which involves clustering stakeholders according to their responsibilities and identifying relevant features. The main contribution of this research lies in enhancing decision-making processes within IoT security management. The proposed stakeholder categorization provides valuable insights into the diverse roles and responsibilities of stakeholders in IoT ecosystems, enabling a better understanding of their interrelationships. This categorization facilitates more effective decision making by considering the specific context and responsibilities of each stakeholder group. Additionally, the study introduces the concept of weighted decision making, incorporating factors such as role and importance. This approach enhances the decision-making process, enabling stakeholders to make more informed and context-aware decisions in the realm of IoT security management. The insights gained from this research have far-reaching implications. Not only will they benefit stakeholders involved in IoT security, but they will also assist policymakers and regulators in developing effective strategies to address the evolving challenges of IoT security.

## 1. Introduction

The Internet of Things (IoT) has experienced rapid growth and is increasingly pervasive in various domains, including healthcare, transportation, manufacturing, and smart homes. This expansion highlights the urgent need to address the security challenges associated with managing the large amount of data generated by IoT devices [1,2]. As the number of connected devices increases, so do the security risks. IoT devices often have vulnerabilities that can be exploited by malicious actors, resulting in privacy breaches, data leaks, device tampering, or even physical harm. Investigating and addressing these security risks is crucial to safeguarding the integrity, confidentiality, and availability of IoT systems. Understanding the roles, responsibilities, and interdependencies of these stakeholders is essential for effective decision making, resource allocation, and risk mitigation strategies. This article’s focus on stakeholder categorization contributes to enhancing the understanding of stakeholder dynamics in IoT security management.

The management of IoT security and clustering stakeholders face several bottlenecks [3,4,5,6], including scalability challenges, heterogeneity and interoperability issues, privacy and data protection concerns, collaboration and communication gaps, adaptability to dynamic IoT environments, and optimizing security measures for resource-constrained IoT devices. To overcome these challenges, it is essential to engage in multidisciplinary research efforts and make advancements in security protocols [5], privacy-enhancing technologies [5], standardization [6,7], collaborative frameworks [7,8], and resource-management techniques [5]. By effectively addressing these bottlenecks, the field of IoT security and stakeholder management can make substantial progress in achieving secure and sustainable IoT deployments.

The paper make a significant contribution to the theoretical understanding of IoT security management by classifying stakeholders based on their roles and responsibilities. This framework provides valuable insights into the diverse stakeholders in IoT ecosystems and their interrelationships. Enhancing decision making by considering factors such as role and importance. Various statistical techniques, including chi-square, F-test, and logistic regression, are employed to validate the research and enhance the understanding of validation methodologies.

The results of this research will also provide valuable guidance for IoT device manufacturers, cloud service providers, and other stakeholders to prioritize and allocate resources to address the most critical security risks and collaborate in building a secure and trustworthy IoT ecosystem. This paper is organized as follows: Section 2 provides background information, Section 3 discusses related work, Section 4 outlines the methodology, Section 5 presents a case study, Section 6 discusses the results and their implications, and Section 7 concludes the paper. Section 8 provides limitations and suggests future work.

## 2. Background

The IoT is a rapidly expanding technology with the potential to transform numerous facets of our daily lives. IoT devices are equipped with sensors and communication capabilities, allowing them to collect and transmit data over the Internet. These devices have utility across a range of applications, including smart residences, industrial automation, and transportation networks [9].

However, the growing popularity and extensive use of IoT devices have also introduced new security challenges. IoT devices often lack proper security measures, rendering them vulnerable to attacks [10,11]. These attacks can range from simple network-based attacks to more sophisticated ones that target the physical devices themselves [11].

The security of the IoT ecosystem is a complex and interdisciplinary domain that combines cybersecurity with various engineering fields, such as mechanical and electrical engineering [12,13]. It goes beyond protecting data, servers, network infrastructure, and information. It also involves the supervision and management of physical systems connected through the Internet, whether in a centralized or distributed manner [14,15].

### 2.1. Taxonomy

**Figure 1:**
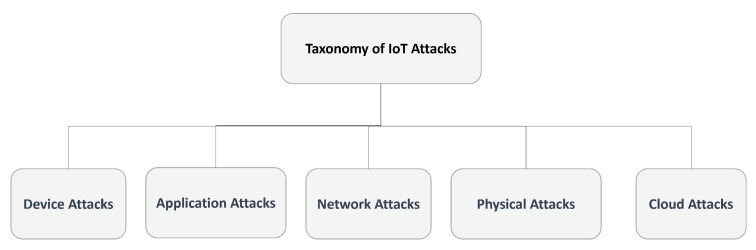
Taxonomy of IoT attacks based on different features.

Different categories of attacks can significantly impact the security of IoT devices and the information they collect and transmit [16]. It is essential for both organizations and individuals to have knowledge about these attack types and implement appropriate measures to protect against them. A classification of IoT attacks is illustrated in Figure 1.

#### 2.1.1. Device Attacks in IoT

Security threats targeted toward specific devices or types of devices exploit vulnerabilities in the hardware or software of the device, potentially causing harm to the device itself or the network it is connected to [17]. These device-specific attacks involve exploiting known vulnerabilities in the device’s operating system, firmware, or hardware, compromising the device through phishing attacks, or even physically tampering with the device [18,19]. As the number of IoT devices continues to grow, it has become crucial for manufacturers to prioritize device security, and users must also take proactive measures to protect their devices [20,21]. This can include keeping software up to date, using strong passwords, and exercising caution when connecting to untrusted networks.

#### 2.1.2. Application Attacks in IoT

Security threats that target the applications and software running on IoT devices exploit vulnerabilities within the applications, including issues within the code or the way the application interacts with other systems [22,23]. Examples of application attacks in the IoT include cross-site scripting [24], SQL injection [18], and buffer overflow attacks [25]. These attacks can compromise the security of the device and potentially grant attackers access to sensitive data or control over the device. To prevent application attacks in the IoT, it is crucial for developers to adhere to secure coding practices, and users should ensure their devices are updated with the latest security patches and software versions. Additionally, employing encryption and authentication technologies can help protect against application attacks in the IoT.

#### 2.1.3. Network Attacks in IoT

Security threats that target the network infrastructure used by IoT devices exploit vulnerabilities within the network itself, potentially compromising the security and functionality of connected devices. Examples of network attacks in the IoT include man-in-the-middle attacks [26], denial-of-service (DoS) attacks [27], and unauthorized access attacks [28]. These attacks can enable attackers to intercept and manipulate data transmitted over the network or disrupt the network, affecting the availability and reliability of connected devices. To prevent network attacks in the IoT, organizations should implement secure network design and deployment practices, such as using secure protocols, firewalls, and access controls. Additionally, regularly monitoring network activity and promptly addressing any security incidents can help mitigate the risk of network attacks in the IoT.

#### 2.1.4. Physical Attacks in IoT

Physical attacks in the context of IoT refer to security threats that involve the physical manipulation of a device [18]. These attacks can range from simple tampering to more sophisticated and malicious activities, including theft or destruction of the device [29,30]. Physical attacks can be particularly detrimental in critical infrastructure systems used in sectors such as healthcare, transportation, or energy production [31]. To prevent physical attacks, it is crucial for manufacturers to prioritize security in the design of their devices, and for users to secure their devices in physically inaccessible locations to unauthorized individuals. Additionally, implementing measures such as secure enclosures, tamper-evident seals, or biometric authentication can help mitigate the risk of physical attacks.

#### 2.1.5. Cloud Attacks in IoT

Security threats targeting IoT devices’ cloud infrastructure and services exploit vulnerabilities in the cloud platform, its applications, or the communication between the cloud and IoT devices [32]. Examples of cloud attacks in IoT include cloud data breaches, server misconfigurations, and unauthorized access to cloud resources [32,33]. These attacks can compromise sensitive data stored in the cloud, disrupt the functioning of connected IoT devices, or grant attackers unauthorized access to cloud resources. To prevent cloud attacks in IoT, organizations should adopt secure cloud deployments and management practices, such as implementing encryption, access controls, and monitoring tools. Regularly updating and patching cloud platforms and applications can also help mitigate the risk of cloud attacks in the context of IoT.

### 2.2. Impact of Attacks

The impact of attacks in the field of IoT security can be substantial, resulting in various consequences, such as financial losses, reputational damage, physical harm, and loss of critical information. Having an understanding of these impacts is crucial for organizations and individuals to prioritize security measures and mitigate the risks associated with IoT attacks. This section discuss the impact of attacks in three specific areas: side-channel attacks (SCA), post-quantum cryptography (PQC), and standardization efforts.

#### 2.2.1. SCAs

SCAs pose a significant threat to IoT security, as they exploit unintended side-channel leakages to extract sensitive information. These attacks can have severe consequences, including the unauthorized disclosure of cryptographic keys and confidential data, thereby compromising the overall security of IoT systems [34]. To mitigate the impact of SCAs, several countermeasures have been developed [35,36,37], such as error detection and correction techniques, redundancy mechanisms, secure implementation practices, and masking techniques that introduce random noise to power traces or resist power analysis.

The combination of Differential Power Analysis (DPA) and Differential Fault Analysis (DFA) attacks poses a significant threat to cryptographic implementations. Attacks that exploit unintended side-channel leakages, such as power consumption, electromagnetic radiation, or timing information, can extract sensitive information from cryptographic implementations [38]. To mitigate the risks associated with these combined attacks, countermeasures such as Threshold Implementations (TI) circuits and error detection schemes are crucial. TI circuits provide built-in security features and tamper-resistant designs, while error detection schemes incorporate redundancy and error-checking mechanisms [39]. These measures enhance the resilience of cryptographic systems and protect against the compromise of sensitive information through fault and power analysis [38,40]. By implementing these countermeasures, the security of cryptographic implementations can be effectively enhanced against combined DPA and DFA attacks.

Field-Programmable Gate Arrays (FPGAs) play a crucial role in implementing cryptographic algorithms for IoT devices. However, the physical characteristics of FPGAs, such as power consumption, electromagnetic radiation, and timing information, can unintentionally leak information about the internal operations and secret keys of the implemented algorithms [41]. SCAs, including power analysis attacks and fault attacks, take advantage of these leakages to extract sensitive information from FPGA-based implementations. Power analysis attacks analyze power consumption patterns to infer secret keys [42], while fault attacks manipulate the FPGA to induce faults and analyze resulting behavior variations [43].

To enhance the security of FPGA-based implementations against SCAs, researchers have been working on countermeasures, including those targeting post-quantum cryptographic algorithms such as Ring-Learning with Errors (Ring-LWEs) [44,45]. These countermeasures aim to mitigate side-channel leakages and protect sensitive information processed by FPGAs. Furthermore, specific fault detection techniques for FPGA platforms have been developed to detect and mitigate the impact of faults in cryptographic algorithms such as Ring-LWEs [46].

#### 2.2.2. PQCs

With the rise of quantum computing, there is a growing concern that traditional cryptographic algorithms, such as Elliptic Curve Cryptography and Rivest–Shamir–Adleman, may be vulnerable to being broken by quantum computers [47,48]. Post-Quantum Cryptography (PQC) aims to address this challenge by providing cryptographic algorithms that are resistant to attacks by quantum computers [6]. The adoption of PQC has implications for security applications across various domains, including IoT [49]. The impact of PQC implementation on IoT security is twofold [6,50,51]. Firstly, the adoption of PQC algorithms requires significant changes in cryptographic protocols and infrastructure. This transition may introduce challenges, such as increased computational and storage requirements for IoT devices, which could potentially affect their performance and resource constraints. Secondly, ensuring compatibility between legacy IoT systems and PQC algorithms is crucial to ensure a seamless transition without compromising security. Efforts are currently underway to standardize PQC algorithms and protocols, aiming to achieve interoperability and widespread adoption. Standardization of PQC is essential in establishing a secure foundation for future IoT deployments, as it enables the development of robust cryptographic systems capable of withstanding attacks from quantum computers.

In the context of embedded systems, including IoT devices, it is crucial to have specific implementations of PQC algorithms that are optimized for ARM Cortex M4 and Cortex-A processors. These processors are widely used in embedded systems due to their low power consumption and cost effectiveness [52,53,54].

Several previous papers have focused on the development and analysis of PQC implementations on ARM processors, specifically the Cortex-M4 and Cortex-A processors. For example, refs. [55,56,57] discusses the implementation of Curve448 and Ed448 algorithms on the Cortex-M4 processor. In [6,58], the focus is on the implementation of the SIKE (Supersingular Isogeny Key Encapsulation) algorithm on the Cortex-M4 processor, with the latest version being SIKE Round 3 [58,59]. Furthermore, ref. [60] explores the implementation of the Kyber post-quantum cryptographic algorithm on 64-Bit ARM Cortex-A processors. Kyber is a lattice-based PQC algorithm.

Fault detection and diagnosis techniques are of paramount importance in ensuring the reliability, integrity, and security of cryptographic algorithms such as the Pomaranch cipher [61], Grostl hash [62], Midori cipher [63], and RECTANGLE cipher [63]. These techniques play a vital role in identifying and mitigating faults that can compromise the functionality and resilience of cryptographic systems. By promptly detecting and addressing faults, these techniques help maintain the effectiveness and robustness of cryptographic algorithms, thus safeguarding sensitive information and providing protection against potential attacks.

#### 2.2.3. Standardization Efforts

Standardization plays a critical role in enhancing IoT security by providing consistent frameworks, protocols, and guidelines for implementing secure systems. The aim of standardization efforts is to establish best practices and promote interoperability, enabling different IoT devices, platforms, and services to seamlessly work together while ensuring security. By defining common security requirements, protocols, and encryption algorithms, standardization efforts help prevent vulnerabilities and ensure the adoption of robust security mechanisms in the IoT ecosystem [10]. Standardization also provides guidelines for secure communication, authentication, access control, and data protection, thereby mitigating the risks associated with IoT attacks [7].

The NIST (National Institute of Standards and Technology) is a U.S. federal agency with the responsibility of promoting and maintaining standards in various fields, including cryptography [64]. In the domain of lightweight cryptography, NIST has actively participted in the standardization process to identify and promote cryptographic algorithms suitable for resource-constrained devices, such as those used in IoT devices and embedded systems. NIST’s efforts in lightweight standardization aim to evaluate and select cryptographic algorithms that offer strong security while requiring minimal computational resources [63]. These algorithms are designed to meet the specific constraints of resource-constrained devices, including low power consumption, limited memory, and processing capabilities [65].

To address the evolving technologies and challenges in IoT security, standardization efforts must encompass areas such as SCAs and PQC, and the specific requirements of embedded systems such as ARM Cortex M4 and Cortex-A implementations. The development and adoption of comprehensive security standards that cover these aspects are crucial for establishing a strong security foundation for IoT devices and systems. By understanding the impact of attacks in the areas of SCAs, PQC, and standardization, stakeholders can effectively develop countermeasures and ensure the security and resilience of IoT ecosystems. This understanding allows for the proactive enhancement of the security posture of IoT systems, protection of sensitive information, and mitigation of risks associated with emerging threats.

Table 1 summarizes various attack types in the field of cybersecurity and provides information on their impact and corresponding countermeasures. The table highlights different categories of attacks, including device attacks, application attacks, network attacks, physical attacks, cloud attacks, SCAs, DFA and Differential Power Analysis (DPA) attacks, and PQC attacks. For each attack category, the table includes specific attack types, the potential impact on security, and recommended countermeasures to mitigate the risks.

## 3. Related Work

This section explores two aspects: clustering stakeholders and feature selection.

### 3.1. Clustering of Stakeholders

Clustering stakeholders is an important task in project management, aiming to group stakeholders based on their needs, preferences, and characteristics. Various clustering techniques have been proposed to enhance stakeholder classification in projects. However, in software projects, stakeholder classification and conflict resolution can be challenging due to the presence of diverse stakeholders with different needs and requirements. Several studies have proposed different approaches to address these challenges. For instance, one study introduced a fuzzy inference system using a selective Bayesian classifier and a dynamic evolving neural-fuzzy inference system to improve stakeholder classification in projects [66]. Another study employed an embedded Markov framework and a mixture of Markov models to cluster career paths in the IT sector [67]. In the context of software-intensive systems, an expert-based and clustering strategy framework was developed to locate and resolve requirements conflicts [68]. Additionally, a framework utilizing text mining and clustering approaches was proposed to enhance the requirement prioritization process in software development projects [69]; while these approaches have demonstrated potential advantages such as improved accuracy and support for decision making, they also have limitations. These limitations include reliance on the performance of clustering algorithms and the need to consider additional variables such as geographical location and employer. Therefore, selecting an appropriate approach requires careful consideration of the specific needs and requirements of the project.

### 3.2. Feature Selection

The emergence of IoT has brought forth new challenges in data analysis and decision making due to the vast amount of data generated by IoT devices. Recent studies have focused on addressing these challenges through feature selection and unsupervised learning methods [70]; while unsupervised learning techniques such as clustering and PCA can effectively identify patterns in IoT data, they may not capture all the relevant information. To improve the accuracy of machine learning classifiers, novel feature selection methods have been proposed. These methods include CorrACC [71], information gain (IG), and gain ratio (GR) [72], which employ different approaches to select relevant features for classification. Additionally, hybrid feature selection strategies that combine filter-based and wrapper methods [73] have been suggested to reduce the size of the feature set and enhance detection accuracy. However, these methods may have limitations, such as increased computational cost or a fixed threshold for feature selection.

The detection of Android malware targeting Android devices is another significant area of research in IoT data analysis [74]. Feature selection techniques have been proven to enhance the accuracy of malware detection classifiers. However, the effectiveness of different techniques varies depending on the learning algorithm employed. Moreover, the study of IoT botnet attacks has prompted the development of feature selection methods to identify bot-based attacks and facilitate post-attack identification. These techniques utilize filter and wrapper methods in combination with machine learning to determine optimal feature sets [75]. The findings suggest that wrapper methods can yield suitable feature sets for all classification challenges, whereas filter methods may not always achieve the same level of effectiveness. Additionally, the study highlights that host-based features are more effective in identifying bot-based attacks, while channel-based features are preferable for post-attack identification.

### 3.3. Previous Studies and Current Work

In the field of IoT security, gaining a comprehensive understanding of stakeholder characteristics is essential for making informed decisions. However, clustering stakeholders with similar features poses a challenge due to the limited research available on this topic specifically in the IoT domain. Existing research on stakeholder clustering primarily focuses on requirements or priorities, overlooking other relevant stakeholder features. Therefore, there is a need for further exploration and investigation in order to develop effective clustering approaches tailored to the unique context of IoT stakeholders.

Additional research is warranted to address the clustering of stakeholders in the IoT security environment, taking into account their responsibilities and characteristics. This approach would be valuable in dealing with the imbalanced nature of IoT data and would provide a deeper comprehension of stakeholder attributes. Such insights are vital for the development and implementation of effective risk management techniques in IoT security. Categories of stakeholders and their unique security requirements can be identified by grouping them according to their responsibilities and features in IoT security. These data can be used to develop specific security procedures and regulations that address the particular needs of each stakeholder group. Further research is needed to devise methods for effectively maintaining the security of IoT systems and devices, which have become increasingly vital in our daily lives. Table 2 provides a summary of the literature review conducted in this study.

## 4. Methodology

**Figure 2:**
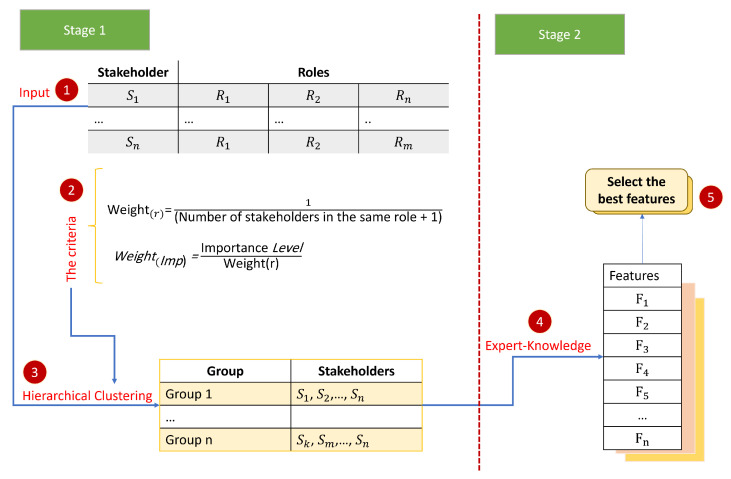
Methodology for smarter decision making and resource allocation.

The proposed methodology for improving IoT security comprises several stages, as depicted in Figure 2. First, stakeholder data are collected and weighted based on their importance. Hierarchical clustering is then used to group stakeholders based on their roles in IoT security. Features are assigned to each cluster utilizing expert knowledge, and the best features are chosen through a voting technique. Finally, the selected features are validated using cross-validation techniques. This approach can lead to enhanced decision making, improved resource allocation, and more effective managing IoT device security.

### 4.1. The First Stage: Clustering Stakeholders

After analyzing stakeholders and their roles, weights need to be assigned to them. The following equations can be used to calculate the weights of the role and importance.

(1) $${\mathrm{Weight}}_{\left(r\right)}=\frac{1}{\mathrm{number}\;\mathrm{of}\;\mathrm{stakeholders}\;\mathrm{in}\;\mathrm{the}\;\mathrm{same}\;\mathrm{role}+1}$$

In Equation (1), the weight of a stakeholder is calculated based on the number of stakeholders in the same role. This equation aims to assign a higher weight to stakeholders who have a smaller number of counterparts in their role. This decision is justified by the assumption that stakeholders with fewer peers hold greater importance and carry a higher level of responsibility in managing IoT security. To determine the number of stakeholders in the same role, a correlation matrix can be utilized. The correlation matrix helps identify the relationships and dependencies between different stakeholder roles.

(2) $${\mathrm{Weight}}_{\left(\mathrm{Imp}\right)}=\frac{\mathrm{importance}\;\mathrm{level}}{Weight_{\left(r\right)}}$$

Equation (2) is used to determine the weight of importance $Weight_{\left(Imp\right)}$ by dividing the importance level of a stakeholder by their corresponding $Weight_{\left(r\right)}$. The purpose of this equation is to prioritize stakeholders based on their level of importance and appropriately distribute their weights. By doing so, stakeholders with higher importance levels receive greater consideration in decision-making processes. To determine the level of importance of stakeholders, a questionnaire was used. The questionnaire results provide information on the relative importance of stakeholders in managing IoT security. Based on the questionnaire responses, weights can be assigned to stakeholders, reflecting their level of importance.

Equations (3) and (4) are normalization equations applied to the weights derived from (1) and (2). The purpose of normalization is to achieve suitable scaling and ensure that the weights fall within a normalized range. By normalizing the weights, they can be compared and consolidated across different stakeholders and importance levels. Normalization is an important step as it allows for the aggregation of normalized weights during subsequent phases of the decision-making process:

(3) $$NormalizeWeight_{\left(r\right)}=\frac{Weight_{\left(r\right)}}{TotalWeights_{\left(r\right)}}$$

(4) $$NormalizeWeight_{\left(Imp\right)}=\frac{Weight_{\left(Imp\right)}}{TotalWeights_{\left(Imp\right)}}$$

After assigning weights to the stakeholders, the next step is to cluster them into groups using hierarchical clustering. Hierarchical clustering involves building a hierarchy of clusters based on the similarity of the data points to one another [76,77]. There are two main approaches to hierarchical clustering: agglomerative and divisive. The divisive strategy starts with all items in one cluster and then repeatedly splits the clusters. On the other hand, the agglomerative approach starts with each object in a distinct cluster and incrementally brings clusters together [76]. In the context of stakeholder identification, hierarchical clustering can be employed to group stakeholders with similar weights into clusters. Ward’s method is a commonly used hierarchical clustering algorithm that aims to minimize the sum of squared distances between the clusters. It is suitable for datasets of various sizes, ranging from small to large [78,79,80]. The equation for Ward’s method is as follows:

(5) $$\Delta D^{2}=D_{ij}^{2}-\frac{D_{i}^{2}+D_{j}^{2}-D_{..}^{2}}{n-2}$$

where $D_{ij}^{2}$ is the squared Euclidean distance between two clusters *i* and *j*, $D_{i}^{2}$ and $D_{j}^{2}$ are the variances of the distances within each cluster, and $D_{..}^{2}$ is the variance of all distances. The parameter *n* is the total number of observations in the two clusters.

Equation (5) quantifies the dissimilarity $\Delta D^{2}$ between two stakeholders by calculating the difference between the squared Euclidean distances $D_{ij}^{2}$ of these stakeholders and the sum of their squared Euclidean distances from all other stakeholders ($D_{i}^{2}$, $D_{j}^{2}$, and $D_{..}^{2}$). This equation is derived from the Euclidean distance formula. Its purpose is to measure the extent to which two stakeholders differ from others in terms of their responsibilities. By evaluating this difference, we can identify stakeholders who exhibit greater distinctiveness and dissimilarity in their roles.

Ward’s method works by iteratively merging the two closest clusters until only one cluster is left. The closeness of clusters is determined by measuring the distance between them using Ward’s equation. After each merge, the method aims to minimize the sum of squared measurements between the clusters, ensuring that the resulting clusters are compact and have similar sizes [78,79].

One advantage of Ward’s method, as described in Algorithm 1, is that it tends to generate clusters with approximately equal variances, which makes it well-suited for datasets with continuous variables that follow a normal distribution. However, the algorithm can be sensitive to outliers and noise present in the data, which may affect the clustering results.

The algorithm presented as Algorithm 1 is referred to as the Ward method with augmented $Weight_{\left(r\right)}$ and $Weight_{\left(imp\right)}$. This algorithm combines the traditional Ward method for hierarchical clustering with the incorporation of weights specifically designed for clustering IoT security stakeholders. The novelty of the algorithm lies in the introduction of these weights, which enables a more refined and contextually sensitive clustering approach for stakeholders. The algorithm incorporates these weights into the computation of the distance matrix using a customized formula that combines the role and importance values along with their corresponding weights. This modification allows the algorithm to effectively capture the unique characteristics and priorities of stakeholders in the IoT security domain.
**Algorithm 1:** The Ward method with weights**Require:** A list of *n* stakeholders *S*, weights $w_{r}$ and $w_{\mathrm{Imp}}$ for the role and importance, respectively.**Ensure:** A clustering of the stakeholders into *k* clusters.1:Compute the distance matrix *D* between all pairs of stakeholders *i* and *j* using the formula: $d\left(i,j\right)=\sqrt{\frac{{\left(w_{r}\left(i\right)\left(r_{i}-r_{j}\right)\right)}^{2}}{w_{r}}+\frac{{\left(w_{\mathrm{Imp}}\left(I_{i}-I_{j}\right)\right)}^{2}}{w_{\mathrm{Imp}}}}$ where $r_{i}$ and $r_{j}$ are the role values for stakeholders *i* and *j*, respectively, and $I_{i}$ and $I_{j}$ are the importance values for stakeholders *i* and *j*, respectively.2:Perform hierarchical clustering on the distance matrix *D* using the Ward method to obtain the dendrogram *T*.3:Cut the dendrogram *T* into *k* clusters using the maximum number of clusters *k* and the criterion maxclust.4:Return the *k* clusters.

### 4.2. The Second Stage: Features Assignment

**Figure 3:**
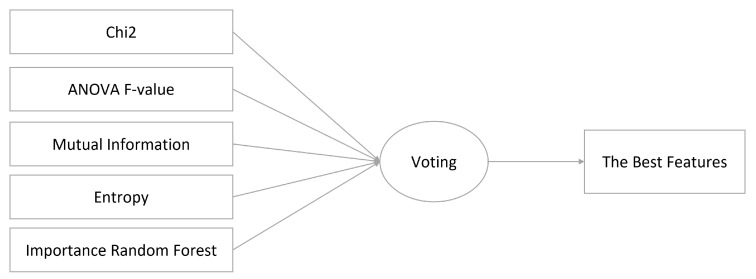
Five models to select the best features from the relevant features.

Data analysis was performed by establishing a connection between roles and features. This mapping facilitated the identification of stakeholders’ specific requirements. The process involved examining the data for patterns and trends, and relevant features were selected for each cluster. To determine the best features from the selected set, various feature selection algorithms were employed, including chi-squared (chi2), Analysis of Variance (ANOVA) F-value (f_classif), mutual information, entropy, and importance random forest (importance_RF). The final subset of features for each cluster was obtained through a voting mechanism, as illustrated in Figure 3.

#### 4.2.1. Chi2

The chi2 statistic is a statistical measure used to assess the association between two categorical variables. It helps determine if there is a significant relationship between the characteristics and the target category [81]. A higher chi-square value indicates a stronger relationship between the variables. The formula for calculating chi-square is as follows:

(6) $$\chi^{2}=\sum_{i=1}^{k}\sum_{j=1}^{m}\frac{{\left(O_{ij}-E_{ij}\right)}^{2}}{E_{ij}}$$

where $\chi^{2}$ is the chi-squared statistic, $O_{ij}$ is the observed frequency of cell (i,j) in a contingency table, $E_{ij}$ is the expected frequency of cell (i,j) assuming independence, *k* is the number of rows in the table, and *m* is the number of columns in the table.

#### 4.2.2. ANOVA F-Value

An ANOVA is a statistical test that compares the means of two or more groups to determine if they are significantly different from each other. It calculates the F-value by evaluating the significance of the differences between the groups, based on the ratio of the between-group variance to the within-group variance [81]. The formula for calculating the F-value for a one-way ANOVA with *k* groups and *n* observations per group is as follows:

(7) $$F=\frac{SS_{between}/\left(k-1\right)}{SS_{within}/\left(nk-k\right)}$$

where *F* is the F-value, $SS_{between}$ is the sum of squares between groups, $SS_{within}$ is the sum of squares within groups, and nk is the total number of observations.

#### 4.2.3. Mutual Information

Mutual information is a measure of the mutual dependence between two variables. It assesses the amount of information one variable provides about the other, and vice versa, and provides a score indicating the strength of the association between them [81]. The calculation of mutual information involves quantifying the extent of information shared between two discrete random variables, represented as *X* and *Y* [81,82]:

(8) $$I\left(X;Y\right)=\sum_{x\in X}\sum_{y\in Y}p\left(x,y\right)\log\frac{p(x,y)}{p(x)p(y)}$$

In the given context, p(x,y) represents the joint probability distribution of random variables *X* and *Y*, while p(x) and p(y) denote the marginal probability distributions of *X* and *Y* individually.

#### 4.2.4. Entropy

Entropy is a measure of the uncertainty or randomness present in a dataset. It quantifies the amount of information contained in a probability distribution and provides a score indicating the level of disorder or unpredictability in the data [82]. The entropy of a discrete probability distribution *P* can be calculated using the following formula:

(9) $$H\left(P\right)=-\sum_{i=1}^{n}p_{i}\log p_{i}$$

where *n* is the number of possible outcomes and $p_{i}$ is the probability of the *i*-th outcome.

#### 4.2.5. Importance Random Forest

Random Forest is an ensemble algorithm that uses decision trees to perform feature selection. It assesses the importance of each feature in the data by measuring its impact on the accuracy of the model. The importance of a feature is ranked, and a score is assigned to indicate its relative importance [83]. In a random forest model, the importance of a feature, denoted as $X_{i}$, can be calculated using the following formula:

(10) $$Importance\left(X_{i}\right)=\frac{1}{N_{tree}}\sum_{j=1}^{N_{tree}}\sum_{t=1}^{N_{node}}\Delta Q_{t,j}\left(X_{i}\right)p_{t,j}$$

where $N_{tree}$ represents the total number of trees present in the ensemble, $N_{node}$ refers to the number of nodes in each individual tree, $\Delta Q_{t,j}\left(X_{i}\right)$ denotes the enhancement in split quality caused by the inclusion of feature $X_{i}$ at node *t* in tree *j*, and $p_{t,j}$ represents the fraction of samples that reach node *t* in tree *j*.

## 5. Case Study on Nine Stakeholders and Two Datasets

This case study focuses on the interactions and viewpoints of nine stakeholders in the field of IoT security. The study utilizes two cybersecurity datasets, namely [84] and UNSW-NB15 [85], to analyze and evaluate various aspects of IoT security.

### 5.1. Step 1: Define Stakeholders and Their Roles

**Figure 4:**
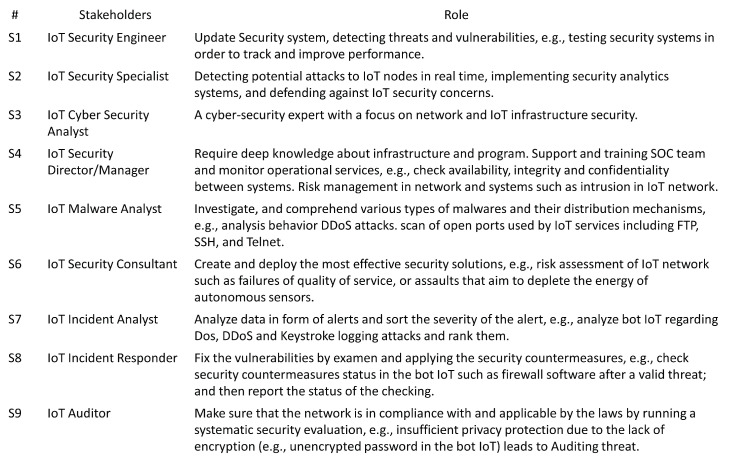
Stakeholders and roles.

Figure 4 shows the first step: defining stakeholders and their roles.

### 5.2. Step 2: Weights and Normalization

During this critical stage, the process entails calculating the weights of the roles and determining the significance of the nine stakeholders. The resulting values are then normalized to ensure consistency and uniformity across the scale.

#### 5.2.1. Weights of Roles

To demonstrate the relationship between stakeholders based on similar roles, a correlation matrix is used. The following equation shows the correlation between $R_{i}$ and $R_{j}$, for i,j=1,2,...,n:

(11) $$Correlation\left(R_{i},R_{j}\right)=\left\{\begin{array}{cc} \; & \;\mathrm{if}\;x=1,\;\mathrm{there}\;\mathrm{is}\;\mathrm{similarity}\\ \; & \;\mathrm{if}\;x=0,\;\mathrm{there}\;\mathrm{is}\;\mathrm{no}\;\mathrm{similarity} \end{array}\right.$$

Table 3 represents the correlation matrix. A value of 1 indicates a high degree of similarity between the two roles, while 0 indicates no similarity between them. These correlation values can be utilized to calculate the weights of the roles, with higher correlation values indicating stronger relationships between roles.

The stakeholders have the following roles: S1, S2, S3, S5, and S6 share the same role. S4 has a unique role, and the remaining stakeholders have the same role.

The weights assigned to different roles are determined by the formula denoted as Equation (1). To calculate the weights, the following approach is employed: for S1, S2, S3, S5, and S6, the weight is obtained by dividing 1 by the sum of 5 and 1, resulting in 0.16. The weight for S4 is calculated by dividing 1 by the sum of 1 and 1, which yields 0.5. Finally, for S7, S8, and S9, the weight is determined by dividing 1 by the sum of 3 and 1, resulting in 0.25.

#### 5.2.2. Weights of Importance

**Figure 5:**
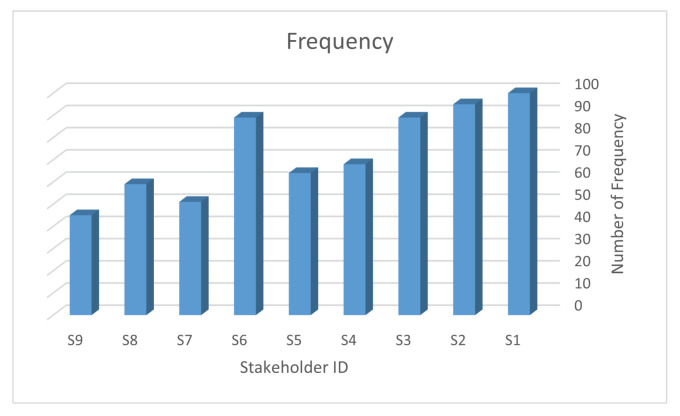
The bar chart shows data for nine stakeholders, reprinted with permission from [86].

In the beginning, a questionnaire was administered to determine the weights of importance for each stakeholder. A total of 124 participants responded and provided their assessments regarding the importance of the 9 stakeholders. Figure 5 presents the results, where a high frequency indicates a high level of importance.

The resulting weights were calculated using Equation (2), based on the values obtained from Figure 5. These weights are summarized in Table 4. To calculate the weights of importance, the values for each role from Figure 5 were divided by their corresponding role weight, which was calculated in the previous Section 5.2.1.

For example, according to Table 4, the weight of the role of the IoT security engineer (S1) is 0.16, and the corresponding value from Figure 5 is 1. Therefore, the weight of importance for S1 is calculated as 1/0.16 = 6.25. Similarly, the weights of importance for the other eight stakeholder roles are calculated, ranging from 1.6 for the auditor (S9) to 6.25 for the IoT security engineer (S1). These weights will be used in subsequent analyses to prioritize and allocate resources to stakeholders based on their importance.

#### 5.2.3. Normalizing Weights

To normalize the weights of role and importance, each weight is divided by the sum of all weights assigned to role and importance. The resulting values represent the normalized weights for each stakeholder role. Table 4 and Table 5 display the weights of each stakeholder role before and after normalization, respectively. These normalized weights will be used in subsequent analyses to prioritize and allocate resources to stakeholders based on their normalized weights.

### 5.3. Step 3: Clustering of Stakeholders

**Figure 6:**
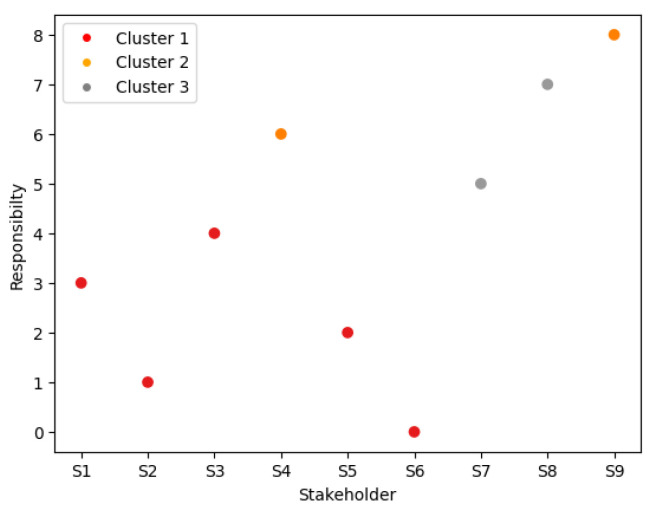
Scatter plot illustrating the clustering of nine stakeholders into three groups.

A scatter plot for dendrograms is a visualization technique used to represent hierarchical clustering. In Figure 6, the data points are plotted on a two-dimensional plane using their respective coordinates obtained from a dendrogram. Each data point represents a leaf node in the dendrogram, and its position in the scatter plot is determined by its distance from other leaf nodes in the dendrogram.

### 5.4. Step 4: Select the Relevant Features

**Figure 7:**
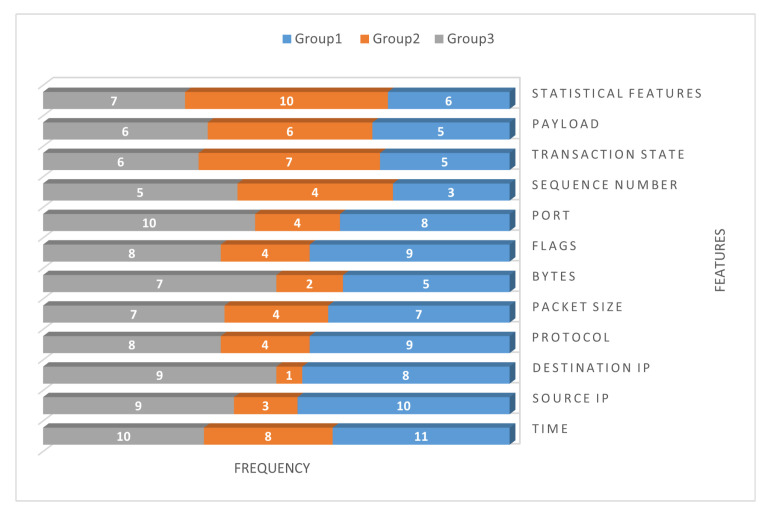
Relevant features for each group based on expert selection.

After Step 3, expert knowledge is applied to select the relevant features. Figure 7 illustrates the features for three groups (clusters), and the relevant feature is defined as any feature with a score of 7 (50%) or higher.

### 5.5. Step 5: Select the Best Features from the Relevant Features

The process is divided into two steps to select the best features for each group. First, five models are used. Second, a voting system is employed.

#### 5.5.1. Five Models on Two Datasets

Based on Table 6 and Table 7, which display the results for the Bot-IoT and UNSW-NB15 datasets, respectively, various feature selection methods were applied. The recorded metrics for each method include classification accuracy, precision, recall, F1 score, and the time taken. The tables are divided into three groups, and each group underwent the same feature selection methods. Within each group, five feature selection methods were employed: chi2, ANOVA F-value, mutual info, entropy, and importance random forest. The tables also provide the selected feature IDs for each method.

#### 5.5.2. Votes to Select the Best Features

Feature selection by vote refers to selecting the features that are most frequently used across all the models. This is typically completed by counting the number of times each feature is utilized in the different models and selecting the ones with the highest counts. The advantage of using this method for feature selection is that it can help identify the most important features that contribute the most to the overall performance of the model. By focusing on these significant features, the model can be simplified and made more efficient without sacrificing predictive accuracy. The results of this approach are presented in Table 8.

### 5.6. Validation of the Results

Cross-validation was employed in every model to ensure the correctness of the features. The best features were determined using votes. The logistic regression model was trained and tested to evaluate the performance of the selected features.

**Figure 8:**
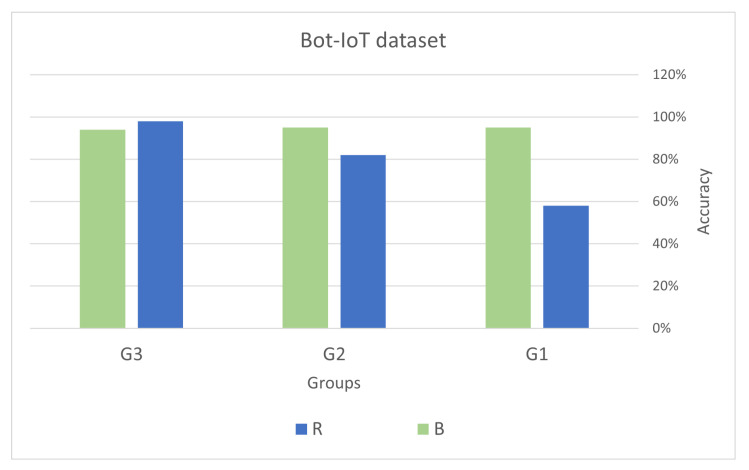
Comparison of the impact of using only the best features vs. other features: analysis of groups G1, G2, and G3 in the Bot_IoT dataset.

**Figure 9:**
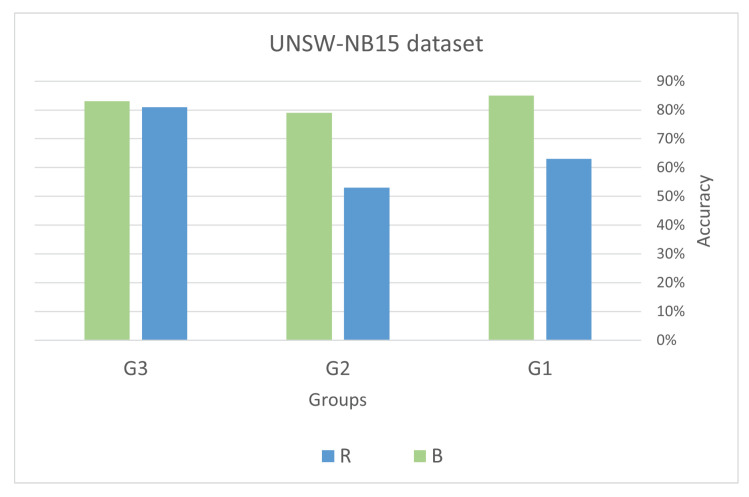
Comparison of the impact of using only the best features vs. other features: analysis of groups G1, G2, and G3 in UNSW-NB15 dataset.

The equation below illustrates the relationship between the sets of features:

(12) R=U−B

where *U* represents the set of all features (universal set), *B* represents the set of best features, and *R* represents the set of remaining features (i.e., those features not selected as the best). The results of the validation process are depicted in Figure 8 and Figure 9. These figures clearly demonstrate that the accuracy of the logistic regression model significantly improved after incorporating the best features. This provides strong evidence that the feature selection process successfully identified the relevant and important features necessary for the classification task.

## 6. Discussion

The results in Table 6 and Table 7 demonstrate the effectiveness of various feature selection methods in improving the classification performance of the Bot-IoT and UNSW-NB15 datasets, respectively. This section discusses the key observations and insights derived from the experimental results. First, it is observed that the chi2 and f_classif methods consistently select the same subset of features, which exhibit similar classification performance in the Bot-IoT dataset, and convergent features in the UNSW-NB15 dataset. This implies that the statistical methods used for feature selection are successful in identifying the relevant features that significantly contribute to classification accuracy.

Second, it was observed that the mutual_info method performed poorly in selecting relevant features for the Bot-IoT dataset, whereas it demonstrated good performance for the UNSW-NB15 dataset. This suggests that the effectiveness of the mutual information method depends on the specific characteristics of the dataset and the relationships between the features. Third, the entropy-based feature selection method resulted in the selection of a higher number of features compared to the other methods for both datasets. This outcome could be attributed to the fact that the entropy-based method takes into account the joint information gain of features, which can introduce redundancy and include irrelevant features. However, despite selecting more features, the chosen ones still achieved comparable classification performance to the other methods.

Fourth, the importance_RF method, which utilizes the feature importance scores of the random forest classifier, identified a smaller subset of features for both datasets. Despite selecting fewer features, this method achieved comparable classification performance to the other methods, indicating its effectiveness as a feature selection technique. Fifth, the time taken for feature selection varies significantly among the different methods. The chi2 and f_classif methods are the fastest, while the mutual_info method is the slowest due to its dependence on the number of features. The entropy-based and importance_RF methods took longer than the statistical methods, but their performance is comparable. Therefore, the choice of feature selection method depends on the available computational resources and the desired classification performance.

In conclusion, it is crucial to emphasize the significance of feature selection in machine learning tasks, as it enhances classification performance and reduces computational expenses [87]. However, the effectiveness of feature selection techniques heavily relies on the specific characteristics of the dataset, the relationships between features, and the classification model employed. Hence, it is essential to evaluate multiple feature selection methods and choose the one that achieves the best classification performance for a given dataset and model. Figure 8 and Figure 9 display the accuracy percentages achieved by different groups (G1, G2, and G3) on the Bot-IoT and UNSW-NB15 datasets using various feature sets.

Both datasets demonstrate that utilizing only the best features leads to higher accuracy compared to using all features except the best one. This indicates that the best features have a substantial impact on accurately predicting the outcome. In the case of UNSW-NB15, G3 achieved the highest accuracy of 83% when using only the best features. However, it is worth noting that the accuracy achieved by using only the *B* is lower than when using *R*, suggesting that other features also contribute significantly to the overall accuracy. In the Bot-IoT dataset, G3 achieved the highest accuracy of 98% when using *R*, while G2 achieved the highest accuracy of 95% when using *B*. This suggests that the importance of the best features is more pronounced for G2 compared to G3. The reason G3 achieved the highest accuracy when using all features except the best one in Bot-IoT could be attributed to their identification and inclusion of the most relevant features specific to their use case. It is possible that the excluded features were not highly relevant for their use case and including them did not significantly improve the model’s accuracy.

On the other hand, G2 achieved the highest accuracy when using only the best features. This indicates that the best features are highly relevant and provide crucial information for their specific use case. The excluded features may have been less important and could have introduced noise or reduced the overall performance of their model. It is worth noting that the results for G1 consistently show lower accuracy compared to the other two groups. This could be due to various factors such as the quality of the data they used, their feature selection process, or their modeling technique.

In the UNSW-NB15 dataset, similar trends are observed where the accuracy is generally higher when using the best features compared to using all features except the best one. However, the overall accuracy is lower than in the Bot-IoT dataset, possibly due to differences in the datasets and the nature of the attacks being detected. These results suggest that the best features are highly relevant and provide significant information for the classification task, and including other less relevant features may not improve the model’s performance. However, it is crucial to carefully select features based on the specific use case and the nature of the analyzed data to achieve the best possible performance.

## 7. Conclusions

In this paper, a novel approach has been presented to enhance decision making in IoT security by clustering stakeholders based on their roles. The clustering of stakeholders offers several potenxtial benefits for organizations and individuals. Accurately identifying and grouping stakeholders in IoT security enables more effective study of relevant features and facilitates data division based on their roles, thereby improving decision making.

Furthermore, novel equations have been developed and utilized to assign weights to stakeholders in the IoT security context. These equations provide a customized approach to stakeholder weighting in the field of IoT security, enhancing the accuracy and effectiveness of decision-making processes.

Through the analysis of stakeholders and expert knowledge, relevant features contributing to effective IoT device security management have been identified. This research not only clusters stakeholders, but also provides a structured framework for categorizing them, enabling a better understanding of the diverse responsibilities and involvement levels of different stakeholder groups.

The findings of this research have implications for stakeholders involved in IoT security, as well as policymakers and regulators. Leveraging insights gained from clustering stakeholders, stakeholders can make more informed decisions, allocate resources effectively, and implement targeted security measures to protect IoT devices. Ultimately, these efforts will contribute to the establishment of a more secure and trustworthy IoT environment that fosters innovation and benefits society as a whole.

In summary, the study enriches existing knowledge in IoT by proposing a novel stakeholder categorization, introducing a weighted decision-making approach, employing diverse statistical validation methods, and offering practical implications for IoT security management. These contributions advance the understanding and practice of IoT security, leading to improved security measures and collaborative efforts within IoT ecosystems.

## 8. Limitations and Future Work

This study’s use of simulated data instead of data from real-world scenarios has certain limitations; while the simulation was carefully designed and validated by experts, it may not fully capture the complexity and variability of actual IoT systems. Future work in the field of clustering stakeholders based on their role in IoT security could explore several directions. One potential area of investigation is expanding the research scope to include additional stakeholder groups, such as service providers and consumers, to gain a more comprehensive understanding of the roles different stakeholders play in IoT security.

Another direction could involve exploring the potential of using the clustering approach to develop new security solutions, such as threat intelligence platforms or risk management tools, specifically tailored to the unique needs of different stakeholder groups. By addressing these areas in future research, it will be possible to further enhance the effectiveness of the clustering approach and provide stakeholders in IoT security with the necessary tools and guidance to better manage security risks and ensure the safe and secure use of connected devices.

## Figures and Tables

**Table 1:** Summary of attack types, impact, and countermeasures in cybersecurity.

Type	Attack	Impact	Countermeasures
Device Attacks [17,18,19,20,21]	Exploiting vulnerabilities in device hardware or software, phishing attacks, physical tampering	Harm to device or network, unauthorized access, data compromise	Regular software updates, strong passwords, cautious network connections
Application Attacks [18,22,23,24,25]	Code vulnerabilities, cross-site scripting, SQL injection, buffer overflow attacks	Compromised device security, data access/control by attackers	Secure coding practices, software patching, encryption, authentication
Network Attacks [26,27,28]	Man-in-the-middle attacks, DoS attacks, unauthorized access attacks	Data interception/manipulation, network disruptions, compromised device functionality	Secure network design, protocols, firewalls, access controls, monitoring
Physical Attacks [18,29,30,31]	Tampering, theft, destruction of devices	Device compromise, data loss, disruption in critical infrastructure systems	Secure device design, physical security measures, enclosures, authentication
Cloud Attacks [32,33]	Cloud data breaches, server misconfigurations, unauthorized access	Data compromise, device disruptions, unauthorized cloud resource access	Secure cloud deployment, encryption, access controls, monitoring, patching
SCA [35,36,37]	Active and passive SCAs, fault attacks, power analysis attacks	Compromise of sensitive information, cryptographic implementations	Error detection/correction, redundancy, secure implementation, masking techniques
DFA and DPA Attacks [38,39,40]	DFA and DPA attacks	Compromise of sensitive information through fault or power analysis	Countermeasures specific to DFA and DPA, such as tamper-resistant designs, error detection, secure implementation
PQC Attacks [6,47,48,49,50,51]	Attacks targeting PQC algorithms and implementations	Compromise of encrypted data, undermining security against quantum computers	Development of PQC algorithms, standardization, secure implementation, compatibility considerations

**Table 2:** Comparison of Studies.

Study	Approach	Limitations
[66] 2018	Clustering stakeholders by using fuzzy inference system	Relies on performance of clustering algorithms
[67] 2022	Clustering job titles by using Markov model	Cannot differentiate between function and responsibility
[68] 2021	Using K-means to cluster conflict resolution	Yet to be validated in other domains
[69] 2020	Clustering stakeholders based on requirement	Does not consider words with similar meaning
[70] 2019	Clustering user behavior using hierarchical clustering and select features using PCA and correlation matrix	Does not capture all relevant information
[71] 2020	Wrapper-based feature selection	Not generalized to other datasets or scenarios; wrapper-based feature selection approaches can also be computationally expensive
[72] 2021	Using IG and GR to detect attacks	The fixed threshold of 50% may not be optimal for all datasets
[73] 2019	Hybrid feature selection: filter-based + wrapper methods	Wrapper methods may be computationally expensive, limiting their applicability in large datasets
[74] 2021	Detecting Android malware by machine learning	Only examines filter-based feature selection on a specific dataset and malware family
[75] 2022	Using feature selection methods for IoT botnet attack detection	Filter techniques do not always obtain the ideal feature set, whereas wrapper methods assure it

**Table 3:** Initial correlation between roles.

	$R_{1}$	$R_{2}$	$R_{3}$	$R_{4}$	$R_{5}$	$R_{6}$	$R_{7}$	$R_{8}$	$R_{9}$
$R_{1}$	1	1	1	0	1	1	0	0	0
$R_{2}$	1	1	1	0	1	1	0	0	0
$R_{3}$	1	1	1	0	1	1	0	0	0
$R_{4}$	0	0	0	1	0	0	0	0	0
$R_{5}$	1	1	1	0	1	1	0	0	0
$R_{6}$	1	1	1	0	1	1	0	0	0
$R_{7}$	0	0	0	0	0	0	1	1	1
$R_{8}$	0	0	0	0	0	0	1	1	1
$R_{9}$	0	0	0	0	0	0	1	1	1

Note: The shaded cells represent the correlations the same input variables *R* in the table.

**Table 4:** The weights of roles and their importance before normalization.

Stakeholders	weight_role	weight_imp
S1	0.16	6.25
S2	0.16	5.937
S3	0.16	5.625
S4	0.5	1.8
S5	0.16	4.25
S6	0.16	3.875
S7	0.25	2.32
S8	0.25	2
S9	0.25	1.6

**Table 5:** The weights of roles and their importance after normalization.

Stakeholders	weight_role	weight_imp
S1	0.024961	0.975039
S2	0.026273	0.973727
S3	0.027682	0.972318
S4	0.217391	0.782609
S5	0.036281	0.963719
S6	0.039702	0.960298
S7	0.097276	0.902724
S8	0.111111	0.888889
S9	0.135135	0.864865

**Table 6:** Performance comparison of feature selection methods on the Bot-IoT dataset.

Group	Method	Feature ID	Accuracy	Precision	Recall	F1 Score	Time
Group 1	chi2	[2, 15, 18, 19, 22]	0.9318	1	0.8637	0.8966	72.1125
	f_classif	[2, 15, 18, 19, 22]	0.9318	1	0.8637	0.8966	74.0828
	mutual_ info	[2, 36,37, 40, 41]	0.9318	1	0.8637	0.8966	795.7370
	entropy	[2, 15, 14, 27, 17, 36, 37, 40, 41,11, 12, 18, 19, 20, 21, 22]	0.9318	1	0.8637	0.8966	252.578
	importance_ RF	[2, 15, 19, 22]	0.9318	1	0.8637	0.8966	1128.947
Group 2	chi2	[2, 15, 18, 19, 22]	0.9318	1	0.8637	0.8966	72.1125
	f_classif	[2, 15, 18, 19, 22]	0.9318	1	0.8637	0.8966	74.0828
	mutual_ info	[2, 36,37, 40, 41]	0.9318	1	0.8637	0.8966	795.737
	entropy	[2, 15, 14, 27, 17, 36, 37, 40, 41, 11, 12, 18, 19, 20, 21, 22]	0.9318	1	0.8637	0.8966	252.578
	importance_ RF	[2, 15, 19, 22]	0.9318	1	0.8637	0.8966	1128.947
Group 3	chi2	[2, 15, 34, 19, 22]	0.9319	1.0000	0.8637	0.8966	94.2569
	f_classif	[2, 15, 34, 19, 22]	0.9319	1.0000	0.8637	0.8966	98.0719
	mutual_ info	[42, 33, 34, 30, 31]	0.8752	0.8003	0.9999	0.8890	1280.683
	entropy	[2, 15, 43 42, 6, 23, 24, 28, 29, 32, 33, 34, 35, 4, 25, 26, 30, 31, 18, 19, 20, 21, 22]	0.9299	0.9960	0.8637	0.8946	348.2347
	importance_ RF	[2, 15, 43, 42, 33, 19]	0.9318	1.0000	0.8637	0.8966	804.577

Note: The Feature ID can be found in the Appendix A (Figure A1).

**Table 7:** Performance comparison of feature selection methods on the UNSW-NB15 dataset.

Group	Method	Feature ID	Accuracy	Precision	Recall	F1 Score	Time
Group 1	chi2	[36, 19, 20, 10, 31]	0.8047	0.7865	0.8648	0.8183	17.6258
	f_classif	[36, 6, 19, 20, 10]	0.7702	0.7845	0.7963	0.7685	23.7648
	mutual_ info	[10, 11, 31, 32, 34]	0.7889	0.7733	0.8541	0.8043	166.4645
	entropy	[36, 6, 14, 19, 20, 40, 10, 11, 31, 32, 33, 34, 35, 27, 28]	0.8426	0.8312	0.8884	0.8513	74.7406
	importance_ RF	[6, 10, 11, 31, 32, 34]	0.7867	0.7733	0.8491	0.8016	207.4284
Group 2	chi2	[10, 11, 31, 7, 24]	0.7911	0.7707	0.8643	0.8086	16.0989
	f_classif	[10, 31, 34, 28, 24]	0.7888	0.7709	0.8590	0.8056	22.0848
	mutual_ info	[10, 11, 7, 23, 24]	0.7900	0.7706	0.8625	0.8073	136.3324
	entropy	[10, 11, 31, 32, 33, 34, 35, 27, 28, 5, 7, 23, 24]	0.7885	0.7747	0.8501	0.8032	79.4134
	importance_ RF	[10, 11, 23, 24]	0.7899	0.7706	0.8624	0.8072	240.6583
Group 3	chi2	[10, 19, 20, 36, 24]	0.8051	0.7865	0.8657	0.8189	24.1376
	f_classif	[10, 19, 20, 6, 24]	0.8012	0.7846	0.8590	0.8146	18.6429
	mutual_ info	[10, 11, 8, 9, 23]	0.7900	0.7708	0.8626	0.8074	225.8134
	entropy	[10, 11, 31, 32, 33, 34, 35, 27, 28, 14, 19, 20, 40, 17, 18, 8, 9, 6, 36, 23, 24]	0.8544	0.8447	0.8926	0.8612	98.0131
	importance_ RF	[10, 11, 34, 18, 8, 9, 23, 24]	0.7890	0.7732	0.8552	0.8049	238.2793

Note: The Feature ID can be found in the Appendix A (Figure A1).

**Table 8:** Results of the vote system from two datasets.

Dataset	Group	Votes			Total
		V5	V4	V3	
Bot_IoT	G1	[2, 34]	[15]	[42, 23, 32, 33, 35]	8
	G2	[2]	[15, 19, 22]	[18]	5
	G3	N/A	[2, 15, 19, 34]	[22, 33, 42]	7
UNSW-NB15	G1	[10]	[31]	[36, 6, 19, 20, 11, 32, 34]	9
	G2	[10,24]	[11]	[31, 7, 23]	6
	G3	[10]	[24]	[20, 11, 23, 9, 8, 19]	8

Note: The notation “V5” indicates that the corresponding feature received votes from all five models, “V4” means the feature received votes from four models, and “V3” means the feature received votes from three models.

**Figure A1:**
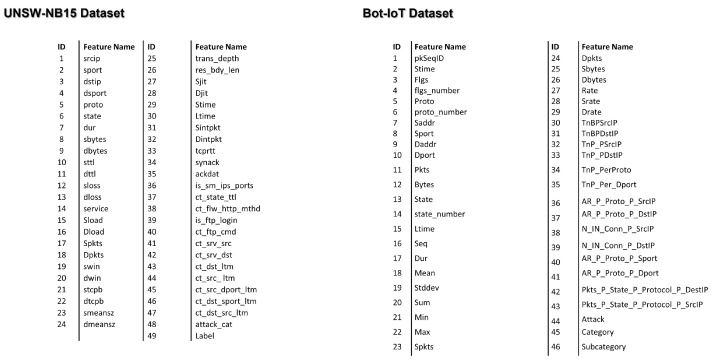
Description of datasets.

## Data Availability

The data presented in this study are available in the article.
